# Address

**Published:** 1890-12

**Authors:** W. H. Sedgwick

**Affiliations:** President


### Address

BY THE PRESIDENT, W. H. SEDGWICK, D.D.S.,

Before the Ohio State Dental Society, Columbus, October 28th, 1890.

*Gentlemen of the Ohio State Dental Society:—*

Again, by the blessing of Divine Providence, the annual cycle has brought us together. As I come before you, to deliver this, my opening address, it is with a keen sense of inability to fulfill my desire or your expectation.

As I look back to the time when I was regularly installed in my honored father's office as a student of dentistry, I am astounded at the advances made in the profession. At that time the best mechanic was regarded as the most successful dentist, and with a few notable exceptions, the preservation of the natural teeth was a secondary object.

The expert extractor who was surgeon enough to sever the *dental ligament* which held the tooth so firmly in the socket was the successful operator. The ambition of my student life was to have a muffle furnace and carve my own blocks for gold mounting.

In my researches for "more light" in dentistry well do I remember visiting a neighboring dentist and seeing over his laboratory door the warning notice "Positively no admittance." During the life time of some who are now within the sound of my voice there were but two practicing dentists in Cincinnati, and the whole State could not claim a half dozen, while there was not one in this capital city of the State, where now blaze forth the pictorial advertisements of a number of self-styled "distinguished" dentists, "Best teeth for $8.00; warranted." Touching upon this subject I can not forbear quoting from Dr. J. B. Wilmot as follows: "The law has its 'shysters,' medicine its 'quacks,' divinity its impostors, but it has remained for dentistry to cheapen itself and depreciate the value of its services to the public. Fancy a lawyer advertising "Best advice only $9.00, poorer quality at $5.00"; or a physician, "Best prescription only 50c; common ones 15c"; or a clergyman, "Best sermons only $5.00 each, and if two be taken on a Sabbath no extra charge made for attending Sunday school in the afternoon," and yet our daily and weekly papers contain scores of this class of dental advertisements to the utter disgust of professional and intelligent men and women."

If we review the period to which I have alluded we find but little done for the advancement and elevation of our profession in the West except isolated individual efforts of a few noble men.

The "itinerant," traveling from town to town was classified as a tinker, a "tooth-carpenter," and occasionally a doctor. Thanks to the indefatigable efforts of industrious men in our ranks we have lived to see dentistry recognized as a learned and scientific profession. The *medical* profession, ever jealous of intruders, has extended to us the hand of fellowship, and welcome us as specialists to its ranks.

What has brought about this rapid change and great advancement in the history of dentistry? I answer the dental colleges, dental literature, and dental societies. It was only in 1840, fifty years ago, that the first regular dental college in the world was established in the city of Baltimore. What pluck, energy, and faith were required by a few noble men to enter upon this new enterprise? Did they, from the summit of Mount Hope, peer into the future, and from its elevated plain see every city of any pretentions supporting a dental college, and the cities and towns of the unknown west maintaining an army of educated dental practitioners? I do not intend to inflict upon you a thread-bare treatise on dental education, yet I would urge upon our colleges the importance of matriculating none but such as give evidence of a good scientific and classical education. Nothing but this higher education prepared Dr. W. D. Miller to take the elevated position in the profession which he now holds. Knowledge enlightens us as to our true condition, makes us ambitious to better it, and clothes us with those social virtues and graces so essential to true manhood; in short, makes us real men.

Let our colleges take no student that has not first had an office pupilage, and practitioners take no student who will not agree to take, at least, a two year's pupilage, and obligate himself to take a regular course of two or three terms, as the case may be, in a regular college of dentistry. To meet my idea of the true specialist, I would have our students take a regular course in a medical college. This gives character and self-confidence to the dentist, and makes him a wise and useful member of our profession, elevating our society, and adding much to our literature.

The journalistic literature of our profession was inaugurated in 1839, by the publication of "*The American Journal of Dental Science*;" the first periodical in the world devoted exclusively to the science of dentistry. To-day our magazines and dental journals form an encylopædia of dental knowledge furnishing information for the inquirer in every branch of our art.

What a history is this to come within the compass of an ordinary life-time? But yesterday a few poor pretenders; to-day a bright array of accomplished, educated practitioners.

Then, Gentlemen of the "OHIO STATE DENTAL SOCIETY," I greet you to-day, with exultation that we have harvested our first fruits, and that the principles of our art have taken the form of a systematic science, and a literature commensurate with the importance of our honorable calling. The transition age is passed; we have reached our majority; the responsibilities and duties of the present era are upon us. I say *us*, for upon the practitioner of to-day depends the advance and attainment accomplished in the future.

I would foster and encourage our State Dental Society, and make it so interesting and instructive that no dentist could afford to absent himself from our annual meetings. So important is associated effort, that now all professions, trades, and callings have their societies, unions, and fraternities. A society may be any thing in result from an honorable association to a selfish conspiracy; accomplishing much good or doing great evil. This society may truly feel proud of what it has accomplished. Intellectually it has been of great advantage; while to some, no doubt, it has been the first school for them to enter; and to-day they are better men and better dentists for the existence of the "Ohio State Dental Society." From a social point of view we have gained much; new friends have been added to our list, and the good accruing therefrom has been far greater than the evil. A demand has been made for more clinics at our annual conventions, and the argument made that many learn more from seeing an operation than from reading even a minute description of the same.

I can not too strongly urge upon you the necessity of the dental law recommended by my worthy predecessor at Cleveland last year. The conditions were not propitious for your committee, appointed a year ago, to make much progress at the session of the legislature last winter. Montebanks, quacks, and impostors are peddling their wares (rubber teeth) from house to house, criminally extracting teeth for the uninformed that should be saved to do good work for years. I would have the strong arm of the law protect the public from these destroyers and mutilators of the "human face divine." I would have the conditions so strict that no one should extract a tooth, or practice dentistry in any branch of the profession without a diploma from a reputable college of dental surgery, or a certificate from the State Board of Examiners, and said diploma or certificate recorded in the clerk's office of the county where the dentist expects to practice; it should be a criminal offence to extract a tooth that could be saved. I would recommend a still more urgent need of district societies, which have not for various reasons, made much headway in our State, from a feeling no doubt, of jealousy or envy existing among a certain class of dentists. It seems difficult to get these dentists into harmony sufficiently to form a society, and these are the very men who need the attrition necessary to bring them up to the standard demanded of the practitioner to-day. "What the circulation of the blood is to the human system, the principle of association is to society; it is the motive, life, and power of its organic being."

I would encourage all to take a part in the discussions and proceedings of the meeting, and especially would I advise our younger members to participate in the deliberations of this session and not leave all the criticism, lectures, and papers to a few.

We are all pupils and all teachers here; take up some subject and give it a special investigation, and give us the benefit of your examination.

Gentlemen of the "Ohio State Dental Society do we as a profession study enough? I answer no! Do we investigate enough? No! Do we write enough? Do we think enough? Do we read enough? Emphatically, no! I would most earnestly recommend that this society inaugurate systematic readings for dental practitioners; a post-graduate course similar to the Chatauqua literary course, such readings directed by eminent specialists in microscopy, biology, bacteriology, physiology, chemistry, etc., and the required monthly readings published in our journals could not but be interesting and instructive. Thus a school would be formed in which all were interested pupils, and a field of research opened to which all inquirers might come, and thus a NATIONAL DENTAL COLLEGE be established for all time. I can not urge too forcibly that all take time to write for publication, interesting and exceptional cases which come under your notice and experience, the modes of treatment and results, whether successful or otherwise, that others may be benefited by your researches. The dentist of to-day is but a school boy, and in the next century I predict more rapid strides will be made in dentistry than in any other profession. I look forward into the future and see a body of intellectual and educated gentlemen with a due appreciation of their high calling; I see a profession exalted by its members; a science so perfected that all things are possible to it; I see a time when the dental pulp will be capped and preserved; when the antiseptics and disinfectants will be so well understood that putrid pulp canals will be scientifically treated, and alveolar abscess aborted; I see a time when oral surgery and hygiene will be referred to the dental practitioner; a time when local anæsthesia will be so perfected that dentistry will be made painless; a time when dentition will be so directed by the skilled dental practitioner that irregularities will be unknown; I see a time when the forceps will be banished from our dental cabinet; a time when our patients will be more careful and fastidious in selecting their dentist than they are in selecting their physician; I see a time when the dentist will command a fair compensation for his work, and his bills be more promptly paid; a time when there will be no combines, trusts, or monopolies, "when all frauds, shams, and impositions shall be swept out as with the besom of destruction and their authors and promotors either converted or brought to shame and confusion."

In conclusion, Gentlemen, may our present session be characterized by harmony, good feeling, and due regard to each other in debates.

"May brotherly love prevail, and every moral and social virtue cement us."

---

DISCUSSION OF THE PRESIDENT'S (DR. SEDGWICK'S) PAPER.

---

Dr. H. A. Smith. A study of the history of the growth and development of dental science in the United States reveals the fact that the inception of its real substantial progress has direct reference to the establishment of the dental college. In advance of that was dental literature. The idea that appears now to have been antecedent to all schemes for organization of any kind took shape in a plan for establishing a dental journal. Two men seem to have conceived this idea at about the same time: Drs. Harris and James Taylor. They, as students, looked upon dentistry as benighted, crude, and they thought that something could be done to establish it on the basis of a special profession. Dr. Harris, while in the South, came in contact with, and formed the acquaintance of, the eminent surgeon, Dr. Marion Sims. He attracted Dr. Sims notice, in the first place, by his manner of treating a case of deformed maxilla, by the use of special appliances. The surgeon became interested in his method, and suggested that it deserved to be published, and urged him to write a description of it. Dr. Harris finally did so. This was his first contribution to a journal; his start in dental journalism. After a time he founded the Baltimore Dental College, independently, as regards the medical profession. Five years later Dr. Taylor, imbued with the same ideas and stimulated by the same ambition, established the Ohio Dental College in Cincinnati.

Dr. Taft. The subjects, literature and dental societies, deserve to be considered in this connection. The tendency of the day is toward lengthening the course of study in dental schools. Back of that is the question of requiring better and more thorough qualifications of matriculates—the question suggested in the paper of requiring as a prerequisite to matriculation a classical education. It does certainly seem that one who proposes to get the greatest possible benefit from the special course prescribed by our dental schools can not be too well qualified through having pursued the general course of training such as classical scools supply. Whether or not the time has come for exacting this as a prerequisite for matriculation in the dental college is a question open for further consideration.

Dr. Smith. Theology is the only profession that exacts this of applicants entering its schools. Such a requirement would exclude three-fourths of our present candidates for graduation in dental schools. But the grade—the class of applicants to our schools—is constantly inproving. The young men who apply are sufficiently well educated for the duties of citizenship, for acquiring a knowledge of all that pertains to the proper and intelligent discharge of ordinary duties outside of the so-called liberal professions. While there may be a larger number of liberally educated men in the law and in medicine, the young men who to-day are turning their attention to dentistry are of a superior class to those of a few years ago. The change in this respect has been very marked since I became a teacher. I think we need not be apprehensive about the question of previous qualification in our students. There is just now such an influx of young men into our colleges that some have expressed a fear that we might, after a while, have too many dentists. If this increase goes on at the present rate, *possibly* in eight or ten years we may have more than are needed. My impression is that we need not be disurbed about that. There is a vast amount of dentistry to be done, and a large number of practitioners is required, and will be required to do it. When the three years requirement obtains there will probably be a falling off in the number of matriculates in our dental colleges. With the increased length of the course will come an increase in the students' fees, which may perhaps unavoidably operate to deter some from entering the colleges.

Dr. Fletcher. Under the general head of education for the student comes the matter of cultivating a habit of observation. Dentists meet together in convention for the purpose of interchanging views, and comparing ideas to the end that they may be mutually benefitted. This presupposes habits of observing, thinking, taking notes, etc., and the result is an accumulation of knowledge, a formulation of ideas which go to make up a vast fund of information from which probably sprang, in the first place, the idea of establishing schools for promulgating that knowledge.

Dr. F. W. Sage. Dr. Sedgwick coming from the old university town of Granville, where every body is steeped in ancient lore, and where Latin, Greek, and the higher mathematics pervade the very atmosphere, naturally attaches considerable importance to the study of the classics. There can be no question that a knowledge of Latin and Greek is of great assistance to the student of medicine, since it aids in an understanding of the use of terms derived from those languages. But after all it is not so much what a man remembers of these studies that assists him in special study, later on. The mental discipline is the great thing. The great requisite for the student of dentistry, medicine, law, or any profession, is a habit of study. A man may have forgotten every line of Latin he ever construed; he may not be able to recall a single proposition in his once familiar Euclid, and still the power of applying himself, and absorbing knowledge from whatever he directs his mind to, remains as *the* important advantage of his ever having studied those branches. It is because so few young matriculates have this kind of mental training that it is found necessary in medical and dental schools to take them through substantially the same course of study and lectures in the second and third years that is laid down for the first year. Few are capable of grasping all that is presented them at once. The majority must have time for absorbing and digesting a part, learning by repetition what at first was missed. In studying they learn how to study, how to observe, how to acquire and fix knowledge in their minds for future use. In the classical school the course of study laid down has reference mainly to mental discipline; in the dental school the student has to deal with facts, practical ideas, methods, which will need to be constantly recalled in his daily practice later on.

Dr. Wilson. I think it is too much to require a classical education at the present time. One of the great faults of students in the old country is, that they devote too much of their time to theory, to the neglect of manual training.

Dr. Taft. The question has been propounded whether it is not desirable to have a division of schools. Each and every college in the country has now a large number of students. Is it desirable to have mechanical schools strictly so called? Should not an effort be made to have pupils trained early in life in the mechanical schools? Men already in active practice are beginning to appeciate the advantage of the post-graduate schools where they can supplement their imperfect knowledge of special branches by placing themselves under the instruction of teachers exceptionally skilled in their various departments.

Dr. Butler. There is a constant demand calling for an adequate supply throughout all departments of life. The public demand better dental service than was possible fifty, or even twenty-five years ago. We are not in danger of having too many dentists. Seven per cent of all our graduates are successful. What is the status of dentistry to-day in England, France, and Germany? With all the abundant supply of their graduates ftom their schools they are still turning their eyes toward America for better service. The demand comes from the people, not from the dental colleges. There are to-day one hundred and seventeen dentists in the city of Cleveland. If the service they rendered was uniformly good, there would be more to be done than this number could accomplish. But it is *not* uniformly well done, and so in the discussion of this question of supplying more dentists, a liberal margin should be allowed for a large per cent. of those already in the field who are practically failures; an account must equally be taken of the number of unfledged students destined to become failures.

Dr. Jennings. I think if the student, when taken into the office, were allowed to work by the side of his preceptor instead of being used as a mere tool, as is often the case; if his attention were called to the various details of cases as they are presented, his pupilage would amount to something. As regards the question of success or failure in practice, it is safe to say that in dentistry no young man can hope to attain to eminence who is not a natural born mechanic. He should have a good eye for colors, so as to distinguish between living and dead dentine, and he must have the artist's instinct, too.

Dr. Taft. Mechanical dentistry is to-day the most neglected branch in our colleges. In the foreign schools this department is better conducted.

